# Ketogenic diet protects MPTP‐induced mouse model of Parkinson's disease via altering gut microbiota and metabolites

**DOI:** 10.1002/mco2.268

**Published:** 2023-05-16

**Authors:** Ziying Jiang, Xinyu Wang, Haoqiang Zhang, Jian Yin, Peiqing Zhao, Qingqing Yin, Zhenfu Wang

**Affiliations:** ^1^ Department of Geriatric Neurology The Second Medical Center & National Clinical Research Center for Geriatric Disease Chinese PLA General Hospital Beijing China; ^2^ Department of Geriatric Neurology Shandong Provincial Hospital Affiliated to Shandong First Medical University Jinan Shandong China; ^3^ Department of Endocrinology The First Affiliated Hospital of USTC Division of Life Sciences and Medicine University of Science and Technology of China Hefei Anhui China; ^4^ Department of Bio‐Medical Diagnostics Suzhou Institute of Biomedical Engineering and Technology Chinese Academy of Sciences Suzhou Jiangsu China; ^5^ Department of Bio‐Medical Diagnostics Jinan Guo Ke Medical Technology Development Co. Ltd. Jinan Shandong China; ^6^ Department of Translational Medical Center Zibo Central Hospital Affiliated to Binzhou Medical University Zibo Shandong China

**Keywords:** gut microbiota, inflammation, ketogenic diet, metabolites, Parkinson's disease

## Abstract

The ketogenic diet (KD) is a low‐carbohydrate, high‐fat regime that is protective against neurodegenerative diseases. However, the impact of KD on Parkinson's disease (PD) and its mechanisms remains unclear. 1‐Methyl‐4‐phenyl‐1,2,3,6‐tetrahydropyridine (MPTP)‐induced mouse model of PD was fed with KD for 8 weeks. Motor function and dopaminergic neurons were evaluated. Inflammation in the brain, plasma, and colon tissue were also measured. Fecal samples were assessed by 16S rDNA gene sequencing and untargeted metabolomics. We found that KD protected motor dysfunction, dopaminergic neuron loss, and inflammation in an MPTP mouse model of PD. 16S rDNA sequencing revealed that MPTP administration significantly increased *Citrobacter*, *Desulfovibrio*, and *Ruminococcus*, and decreased *Dubosiella*, whereas KD treatment reversed the dysbiosis. Meanwhile, KD regulated the MPTP‐induced histamine, N‐acetylputrescine, d‐aspartic acid, and other metabolites. Fecal microbiota transplantation using feces from the KD‐treated mice attenuated the motor function impairment and dopaminergic neuron loss in antibiotic‐pretreated PD mice. Our current study demonstrates that KD played a neuroprotective role in the MPTP mouse model of PD through the diet–gut microbiota–brain axis, which may involve inflammation in the brain and colon. However, future research is warranted to explore the explicit anti‐inflammatory mechanisms of the gut–brain axis in PD models fed with KD.

## Graphical abstract

Microbiota‐targeted dietary intervention is an emerging treatment for Parkinson's disease (PD). We demonstrate ketogenic diet (KD) is different from normal control diets in that they alter the gut microbiota and metabolites in MPTP‐induced PD mice. KD protects against motor dysfunction, loss of dopaminergic neurons, and inflammation in PD mice through the diet–gut microbiota–brain axis (drawn with Science Slides and Power Point).

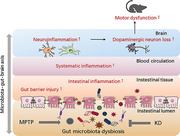

## 1 INTRODUCTION

As one of the most common progressive movement disorders, Parkinson's disease (PD) is characterized by rigidity, resting tremor and bradykinesia.^1^ The core pathological definitions are dopaminergic neuron degeneration or loss in substantia nigra (SN) in combination with α‐synuclein (αSyn) aggregation, regarded as Lewy Bodies (LBs) in the neurons.^2^ The annual incidence rates of PD range from five per 100,000 people to over 35 per 100,000 people, and the prevalence range from 100 per 100,000 people to 300 per 100,000 people.^3^, ^4^ The incidence and prevalence increase sharply with age. Despite the serious public health concern that it poses, only a few medical treatments have been approved for PD, and these work to control symptoms rather than slow the progression of the disease.^5^, ^6^, ^7^

It has been widely recognized the presence of the microbiota–gut–brain axis that significantly affected central nervous system diseases through direct neural, neuroendocrine, and immunological mechanisms. Interestingly, the nonmotor symptoms, especially gastrointestinal dysfunction, including defecatory dysfunction and constipation frequently precede motor symptoms in PD patients, which indicates gut microbial dysbiosis also participates in the development of PD.^8^ ^,‐^ ^9^ A study showed that antibiotic treatment alleviated motor dysfunction and microglia activation, and αSyn pathology in the αSyn‐overexpressing mouse. Oral administration of PD‐derived microbiota induced motor dysfunction, microglia activation, and αSyn pathology in germ‐free mice.^10^ Increased *Akkermansia* and decreased *Faecalibacterium* and *Roseburia* might suppress the production of butyrate and other beneficial short‐chain fatty acids, leading to loss of gut barrier function, systemic inflammation, and abnormal aggregation of αSyn fibrils.^11^, ^12^ Thus, gut microbiota and metabolites participate in the pathomechanism of PD. Microbiota‐targeted interventions, including diet intervention, probiotics, antibiotics and fecal microbiota transplantation (FMT), are considered promising approaches to prevent and treat PD.^13^, ^14^

The ketogenic diet (KD) is a low‐carbohydrate, high‐fat, and moderate‐protein alimentary regimen that achieves nutritional ketosis through increasing the levels of ketone bodies (KBs), mainly β‐hydroxybutyrate (BHB), in the circulatory system.^15^ KD was initially well known as a treatment for drug‐resistant epilepsy in children in the 1920s. KD interventions have recently been reported to play beneficial roles in cancers, obesity, diabetes as well as neurological diseases, including ischemic stroke, amyotrophic lateral sclerosis, and Alzheimer's disease (AD).^16^, ^17^, ^18^, ^19^, ^20^ KD exerts antioxidative and anti‐inflammatory effects and regulates nervous system autoimmunity to prolong lifespan and slows the progression of age‐related diseases.^21^, ^22^ Considering gut microbiota is the pivotal intermediary between diet interventions and host health, the neuroprotective role of KD might be mediated by gut microbiota.^23^, ^24^ A randomized pilot study suggested a modified Mediterranean‐ketogenic diet remodeled gut microbiome and metabolites related to the AD biomarkers in cerebrospinal fluid.^25^ Another study demonstrated KD played the antiseizure role mediated by altering gut microbiome, systemic amino acid γ‐glutamylation, and hippocampal γ‐aminobutyric acid (GABA)/glutamate in mouse models of epilepsy.^26^ In addition, emerging evidence reveals that KD intervention or exogenous KB supplements also improve the mouse model of PD by inhibiting neuroinflammation.^27^, ^28^ Nevertheless, few studies have probed into the interaction between KD intervention and gut microbiota–brain axis in a mouse model of PD.

In the current study, we administrated 1‐methyl‐4‐phenyl‐1,2,3,6‐tetrahydropyridine (MPTP), a toxic substance resulting in loss of dopaminergic neurons in striatum and SN, to develop the common animal model of PD. Our objective was to evaluate the effect of KD on MPTP‐induced mice and to clarify the molecular mechanisms of the diet–gut microbiota–brain axis in the pathologic processes of PD. We found an 8‐week KD intervention alleviated motor dysfunction and dopaminergic neuron damage in SN and inflammation in MPTP male mouse model of PD. Moreover, we used 16S rDNA gene sequencing and ultra‐high‐performance liquid chromatography‐triple/time‐of‐flight mass spectrometry (UHPLC‐Q‐TOF‐MS/MS) to characterize gut microbiota and metabolic alterations after the KD intervention. Finally, we administered FMT using feces from the KD‐treated mice in the antibiotic‐pretreated mice of PD to verify the neuroprotective function of KD‐remodeled gut flora. In general, our findings revealed that KD intervention protected the MPTP mouse model of PD through a diet–gut microbiota–brain axis.

## 2 RESULTS

### 2.1 KD attenuated motor dysfunction in MPTP‐intoxicated mice

**FIGURE 1:**
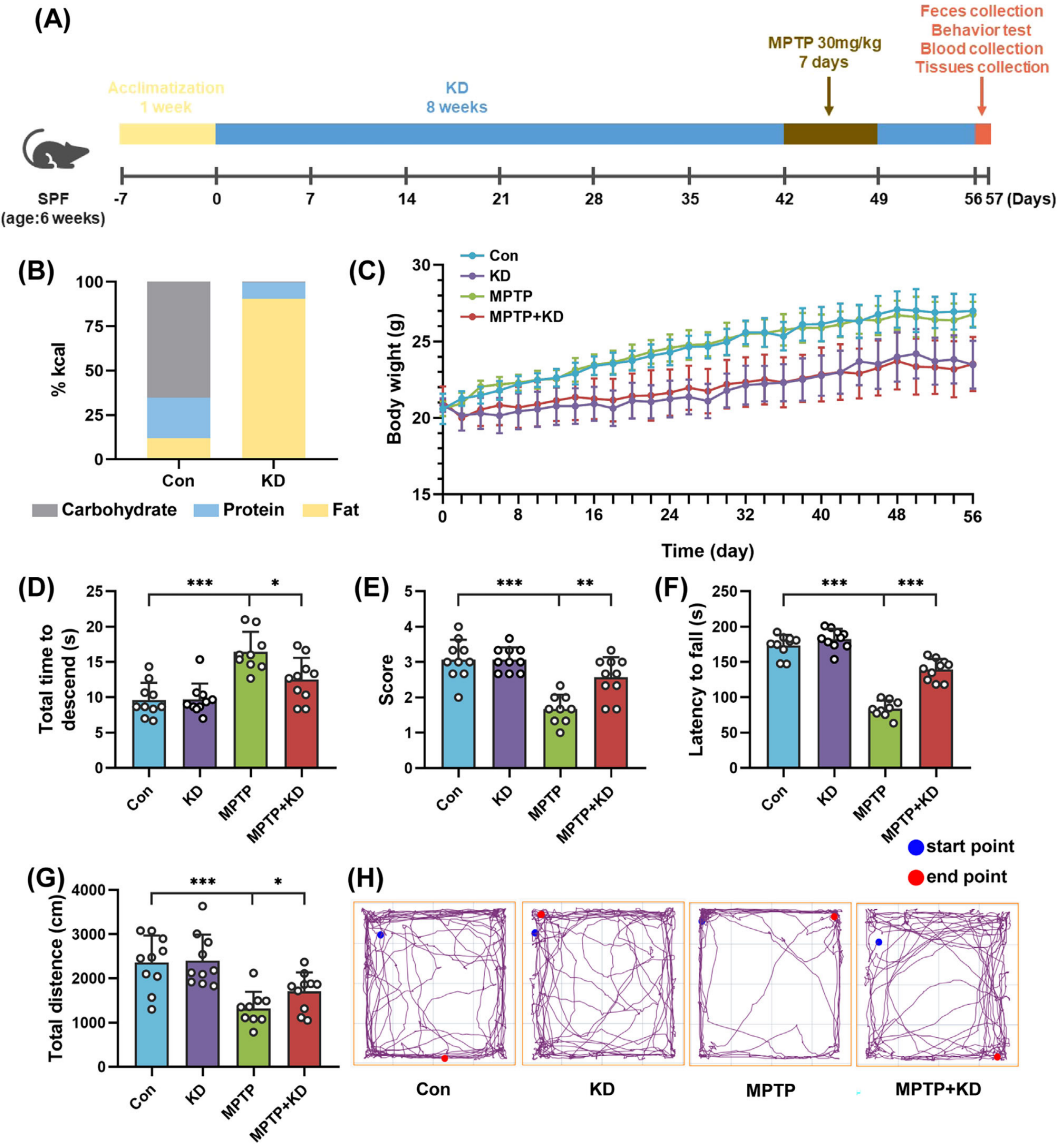
KD attenuated motor dysfunction in MPTP‐intoxicated mice. (A) Schematic representation of study design. (B) Macronutrient composition of diets. (C) Body weight in mice for 8 weeks. (D) Pole test (MPTP, *F*_1, 35_ = 226.253, *p* < 0.001; KD, *F*_1, 35_ = 35.951, *p* = 0.030; MPTP × KD interaction, *F*_1, 35_ = 5.648, *p* = 0.023), (E) traction test (MPTP, *F*_1, 35_ = 37.736, *p* < 0.001; KD, *F*_1, 35_ = 8.467, *p* = 0.006; MPTP × KD interaction, *F*_1, 35_ = 8.467, *p* = 0.006), (F) rotarod test (MPTP, *F*_1, 35_ = 216.763, *p* < 0.001; KD, *F*_1, 35_ = 51.459, *p* < 0.001; MPTP × KD interaction, *F*_1, 35_ = 26.042, *p* < 0.001), and (G, H) open field test (MPTP, *F*_1, 35_ = 27.785, *p* < 0.001; KD, *F*_1, 35_ = 1.726, *p* = 0.198; MPTP × KD interaction, *F*_1, 35_ = 1.139, *p* = 0.293). Data were expressed as mean ± SD, Con group (*n* = 10), KD group (*n* = 10), MPTP group (*n* = 9), MPTP + KD group (*n* = 10), two‐way ANOVA. *p < 0.05, **p < 0.01, ***p < 0.001, and ns indicates not significant.

Mice were fed the normal control diet or KD for 8 weeks (Figures 1A and B). There was no significant difference in weight gain, food intake, and daily caloric intake in the Con and MPTP groups during the 8‐week dietary intervention (Figures 1C and S1A–C). Compared with the normal diet, KD significantly decreased weight gain in mice (Figures 1C and S1A) without altering the daily caloric intake (Figure S1C). Because of the different energy densities of normal control diet or KD, there was a trend toward decreased food intake in KD mice compared with normal diet mice (Figure S1B). After 8 weeks of feeding, KD substantially raised plasma BHB levels compared with normal control diet (Figure S1D), consistent with BHB concentrations in striatum (Figure S1E).

Moreover, the impact of KD on motor performance in mice was evaluated with multiple behavioral tests. Compared with the Con group, the MPTP group showed poorer motor deficits, such as prolonged time to climb down the pole (Figure 1D), reduced scores of traction test (Figure 1E), increased latency to fall from the rotarod (Figure 1F), and decreased total distance traveled (Figures 1G and H). Nevertheless, KD intervention significantly improved motor deficits, such as shortened time to climb down the pole (Figure 1D), increased scores of traction test (Figure 1E), decreased latency to fall from the rotarod (Figure 1F), and increased total distance traveled (Figures 1G and H) compared with the MPTP group. Overall, these findings suggested 8 week KD increased the levels of BHB in plasma and brain and then improved the motor function impairment in the MPTP mouse model of PD.

### 2.2 KD improved dopaminergic neuron loss and inflammation in MPTP‐intoxicated mice

**FIGURE 2:**
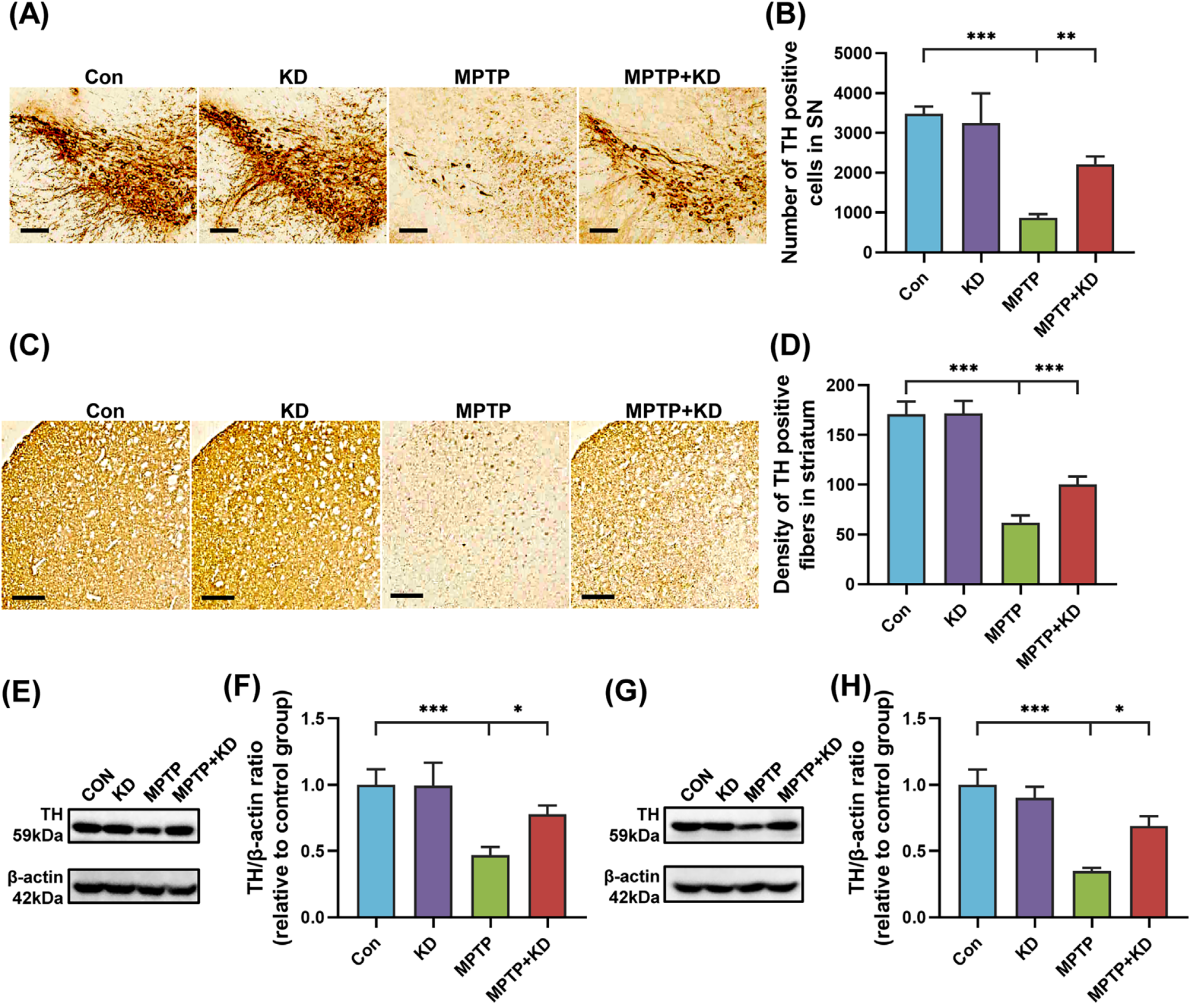
KD improved dopaminergic neuron loss in MPTP‐intoxicated mice. (A) Immunohistochemical staining and (B) quantitative analysis of TH in SN (MPTP, *F*_1, 16_ = 393.354, *p* < 0.001; KD, *F*_1, 16_ = 41.944, *p* < 0.001; MPTP × KD interaction, *F*_1, 16_ = 58.912, *p* < 0.001), scale bars = 100 μm. (C) Immunohistochemical staining and (D) quantitative analysis of TH in striatum (MPTP, *F*_1, 16_ = 371.678, *p* < 0.001; KD, *F*_1, 16_ = 17.550, *p* = 0.001; MPTP × KD interaction, *F*_1, 16_ = 16.492, *p* = 0.001), scale bars = 200 μm. (E) Representative western blot bands and (F) density analysis of TH in SN (MPTP, *F*_1, 8_ = 32.553, *p* < 0.001; KD, *F*_1, 8_ = 5.345, *p* = 0.049; MPTP × KD interaction, *F*_1, 8_ = 5.799, *p* = 0.043). (G) Representative western blot bands and (H) density analysis of TH in striatum (MPTP, *F*_1, 8_ = 152.327, *p* < 0.001; KD, *F*_1, 8_ = 10.116, *p* = 0.013; MPTP × KD interaction, *F*_1, 8_ = 0.678, *p* = 0.434). *p < 0.05, **p < 0.01, ***p < 0.001. Data were expressed as means ± SD, *n* = 5 mice per group, two‐way ANOVA.

**FIGURE 3:**
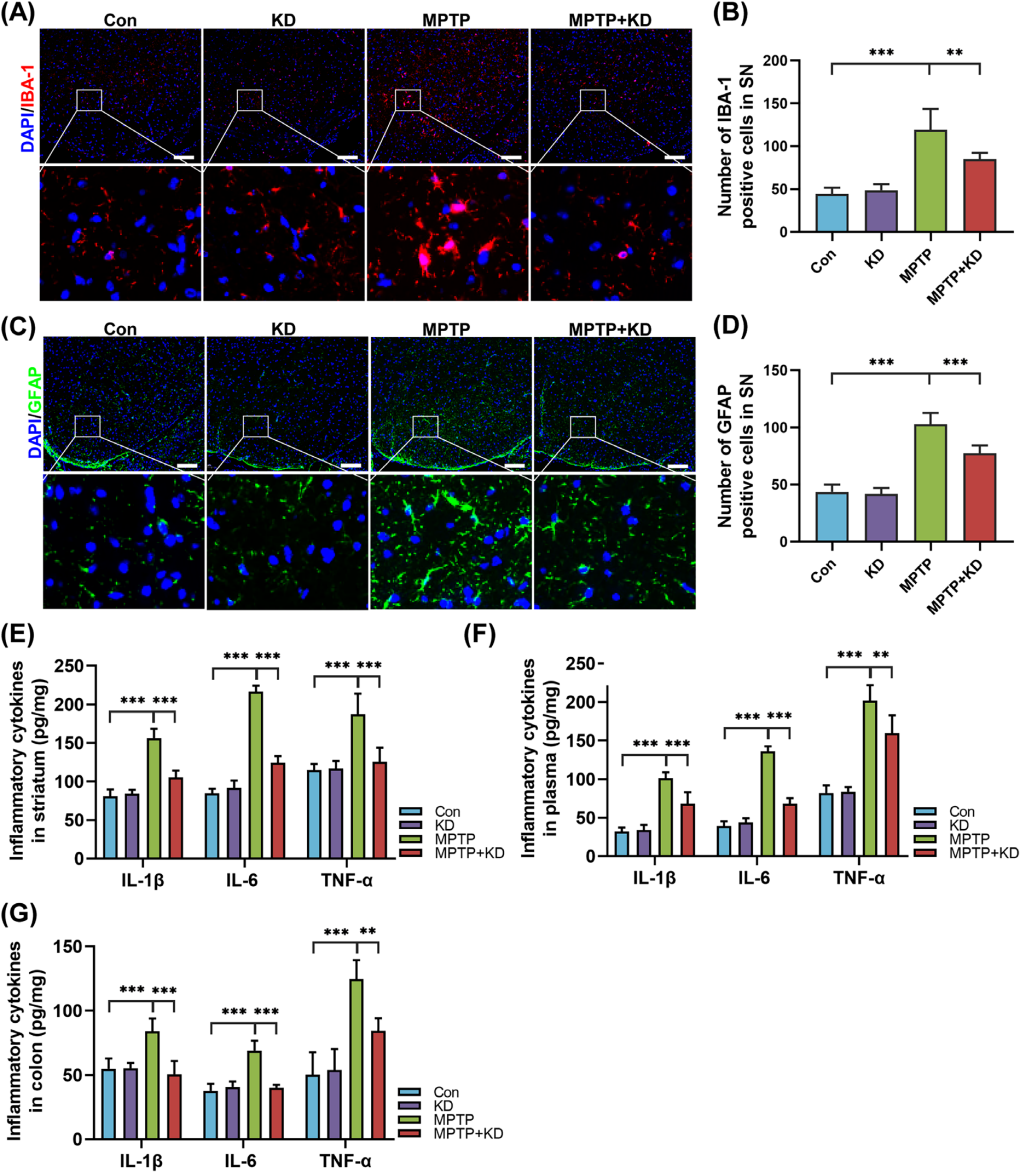
KD inhibited inflammation in MPTP‐intoxicated mice. (A) Immunofluorescent staining and (B) quantitative analysis of IBA‐1 in SN (MPTP, *F*_1, 16_ = 83.056, *p* < 0.001; KD, *F*_1, 16_ = 6.045, *p* = 0.026; MPTP × KD interaction, *F*_1, 16_ = 10.321, *p* = 0.005), scale bars = 100 μm. (C) Immunofluorescent staining and (D) quantitative analysis of GFAP in SN (MPTP, *F*_1, 16_ = 212.963, *p* < 0.001; KD, *F*_1, 16_ = 17.532, *p* = 0.001; MPTP × KD interaction, *F*_1, 16_ = 13.198, *p* = 0.002), scale bars = 100 μm. ELISA analysis of protein expression of IL‐1β, IL‐6, and TNF‐α in (E) striatum (IL‐1β, MPTP, *F*_1, 16_ = 146.549, *p* < 0.001; KD, *F*_1, 16_ = 35.510, *p* < 0.001; MPTP × KD interaction, *F*_1, 16_ = 46.359, *p* < 0.001; IL‐6, MPTP, *F*_1, 16_ = 521.251, *p* < 0.001; KD, *F*_1, 16_ = 139.079, *p* < 0.001; MPTP × KD interaction, *F*_1, 16_ = 190.141, *p* < 0.001; TNF‐α, MPTP, *F*_1, 16_ = 27.183, *p* < 0.001; KD, *F*_1, 16_ = 14.657, *p* = 0.001; MPTP × KD interaction, *F*_1, 16_ = 16.732, *p* = 0.001), (F) plasma (IL‐1β, MPTP, *F*_1, 16_ = 150.117, *p* < 0.001; KD, *F*_1, 16_ = 14.118, *p* = 0.002; MPTP × KD interaction, *F*_1, 16_ = 17.251, *p* = 0.001; IL‐6, MPTP, *F*_1, 16_ = 455.471, *p* < 0.001; KD, *F*_1, 16_ = 123.261, *p* < 0.001; MPTP × KD interaction, *F*_1, 16_ = 161.009, *p* < 0.001; TNF‐α, MPTP, *F*_1, 16_ = 175.441, *p* < 0.001; KD, *F*_1, 16_ = 7.711, *p* = 0.013; MPTP × KD interaction, *F*_1, 16_ = 8.778, *p* = 0.009), (G) colon (IL‐1β, MPTP, *F*_1, 16_ = 10.366, *p* = 0.005; KD, *F*_1, 16_ = 19.030, *p* < 0.001; MPTP × KD interaction, *F*_1, 16_ = 20.198, *p* < 0.001; IL‐6, MPTP, *F*_1, 16_ = 42.903, *p* < 0.001; KD, *F*_1, 16_ = 29.773, *p* < 0.001; MPTP × KD interaction, *F*_1, 16_ = 45.764, *p* < 0.001; TNF‐α, MPTP, *F*_1, 16_ = 62.312, *p* < 0.001; KD, *F*_1, 16_ = 7.639, *p* = 0.014; MPTP × KD interaction, *F*_1, 16_ = 11.068, *p* = 0.004). *p < 0.05, **p < 0.01, ***p < 0.001. Data were expressed as means ± SD, *n* = 5 mice per group, two‐way ANOVA.

Immunohistochemistry and immunoblotting for tyrosine hydroxylase (TH, a vital enzyme in dopamine synthesis) in SN and striatum were performed to detect the dopaminergic neurons in PD mice. The data revealed TH‐positive cells were obviously reduced in SN of the MPTP group compared with the Con group (Figures 2A and B). In contrast, KD partially reversed the TH‐positive cell reduction in SN (Figures 2A and B). Similarly, the TH‐positive fibers density in striatum was reduced after MPTP exposure, whereas KD restored the loss of TH‐positive fibers (Figures 2C and D). Consistent with immunohistochemistry results, KD also promoted TH expression in SN and striatum of MPTP group measured by immunoblotting (Figures 2E–H). Glial cells, including microglia stained by ionized calcium binding adaptor molecule 1 (IBA‐1; sensitive marker of microglia) and astrocytes stained by glial fibrillary acidic protein (GFAP; sensitive marker of astrocyte) were markedly activated by MPTP (Figures 3A–D). However, KD attenuated the glial activation in the MPTP mouse model of PD (Figures 3A–D). Moreover, we evaluated the inflammatory levels in brain, plasma, and colon using enzyme‐linked immunosorbent assay (ELISA) analysis. The results indicated the tumor necrosis factor (TNF)‐α, interleukin (IL)‐1β and IL‐6 levels in striatum, plasma, and colon were all significantly elevated in the MPTP group (Figures 3E–G). However, the MPTP + KD group had relatively lower inflammatory levels in brain, plasma, and colon compared with the MPTP group (Figures 3E–G). Collectively, these data suggested KD contributed to ameliorating PD‐associated dopaminergic neurotoxicity and inflammatory conditions in MPTP‐intoxicated mice.

### 2.3 KD restructured gut microbiota in MPTP‐intoxicated mice

**FIGURE 4:**
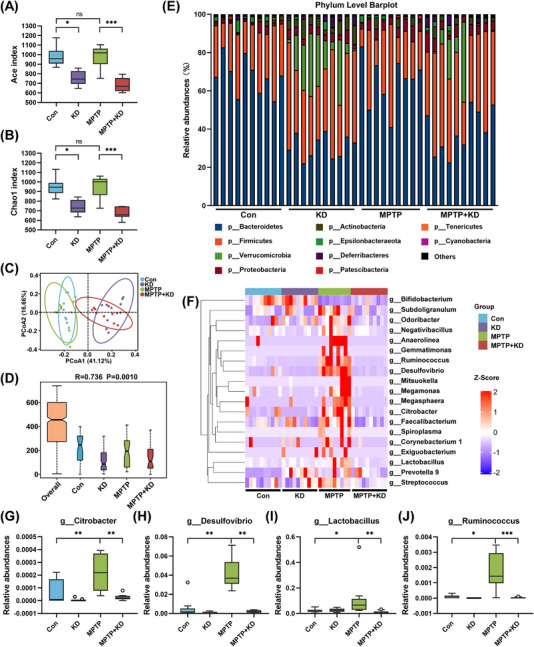
KD restructured gut microbiota in MPTP‐intoxicated mice. Analysis of alpha diversity of intestinal microbiota in mice by (A) Ace index and (B) Chao1 index. Analysis of beta diversity of intestinal microbiota in mice based on (C) PCoA and (D) ANOSIM analysis. (E) Barplot of relative abundance of different groups at the phylum level. (F) Heatmap of relative abundance of different groups at the genus level. Relative abundance of (G) g_*Citrobacter*, (H) g_*Desulfovibrio*, (I) g_*Lactobacillus*, and (J) g_*Ruminococcus* significantly changed among four different groups. Con group (*n* = 10), KD group (*n* = 10), MPTP group (*n* = 9), MPTP + KD group (*n* = 10). *p < 0.05, **p < 0.01, ***p < 0.001, and ns indicates not significant.

Previous accumulating research found intestinal dysbiosis and abnormal metabolites affected the onset and development of PD.^10^, ^13^ Given that our current study found KD suppressed the inflammatory reaction in colon, we further investigated the composition of gut microbiota in MPTP‐induced mice fed with KD using 16S rDNA gene sequencing and bioinformatic analysis. As shown in Figures 4A and B, KD remarkably led to decreased community richness and alpha diversity of gut microbiota species (Ace index and Chao1 index) compared with normal control diet. MPTP slightly increased the Ace index and Chao1 index compared with the Con group, while the alternation was not significant (Figures 4A and B). In addition, beta diversity by principal coordinate analysis (PCoA) demonstrated an obvious distinction of the gut microbiota among the four groups (Figure 4C). Analysis of similarity test (ANOSIM) showed the gut microbial communities were varying among the four groups (Figure 4D), which suggested KD changed the beta diversity.

Based on the taxonomic classification analysis, fecal microbial composition across all samples was illustrated at the phylum (Figure 4E) and family (Figure S2A) levels, respectively. The heatmap exhibited species with differences in relative abundances at the genus level (Figure 4F). Specifically, compared with the Con group, *Citrobacter*, *Desulfovibrio*, *Lactobacillus*, and *Ruminococcus* showed higher relative abundances at the genus level in the MPTP group, whereas KD obviously reduced these relative abundances (Figures 4G–J). Higher relative abundances of *Defluviitaleaceae* and *Lactobacillaceae* were observed in the MPTP group at the family level, while KD significantly decreased these relative abundances (Figures S2B and C). We also displayed other clear alterations for relative abundance of gut microbiota at different taxon levels, although the alternations were partly not significant (Figures S2D–I).

The function of gut microbiota was predicted by the functional analysis based on Kyoto Encyclopedia of Genes and Genomes (KEGG) pathway enrichment and 31 functional pathways were identified (Figure S3). Replication and repair, immune system diseases, and enzyme families were enriched in the Con group. Metabolism, cellular processes and signaling, signal transduction, membrane transport, and other pathways were enriched in the KD group. Neurodegenerative diseases, endocrine system, cell growth and death, metabolic diseases, and other pathways were enriched in the MPTP group. Nervous system was enriched in the MPTP + KD group. Collectively, our results revealed that KD regulated microbiota dysbiosis to protect the MPTP‐intoxicated male mice.

### 2.4 KD altered fecal metabolites in MPTP‐induced PD mice

**FIGURE 5:**
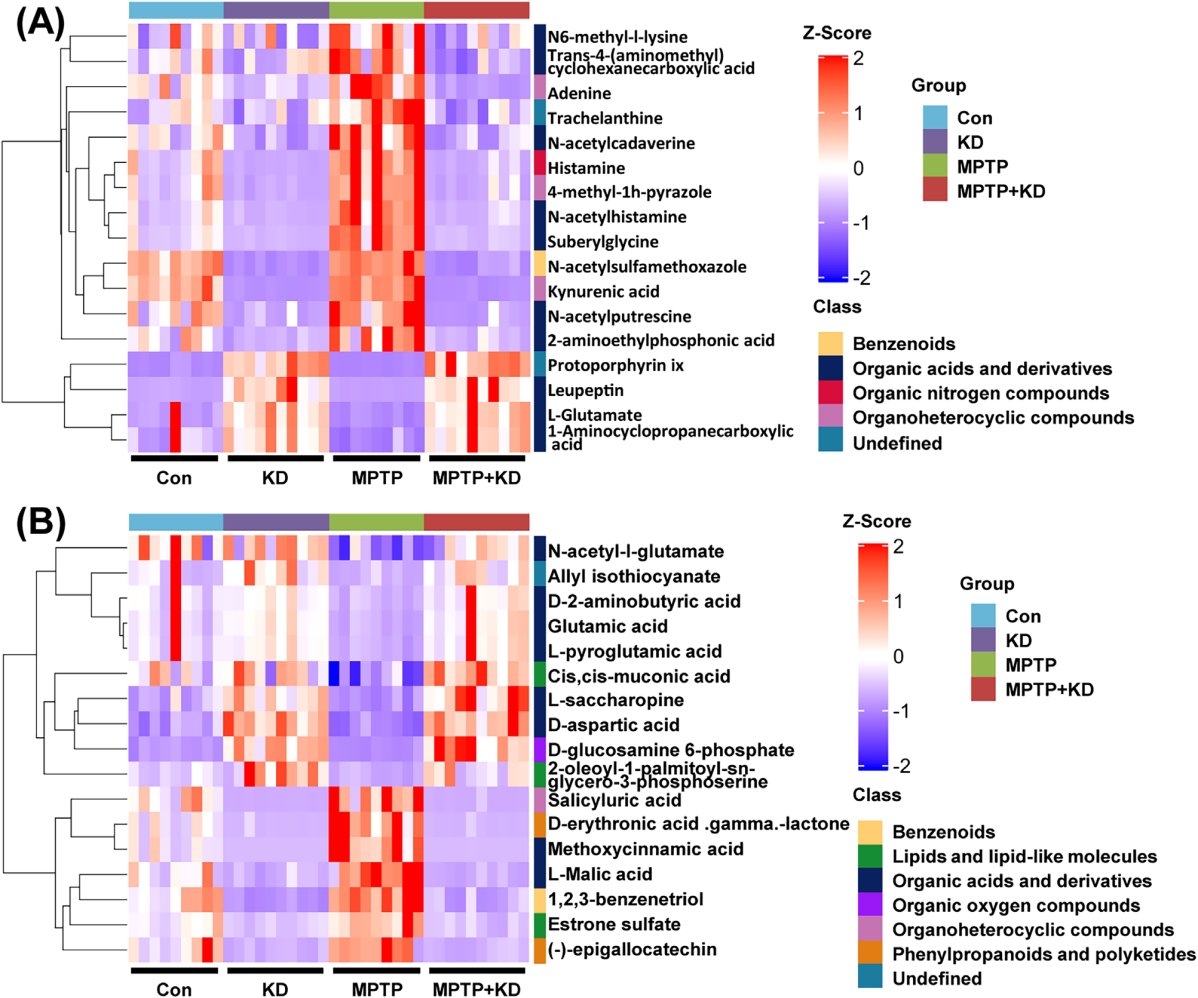
KD altered fecal metabolites in MPTP‐induced PD mice. Heatmap of relative abundance of metabolites among four groups in (A) positive and (B) negative ion mode. Con group (*n* = 9), KD group (*n* = 10), MPTP group (*n* = 9), MPTP + KD group (*n* = 10).

**FIGURE 6:**
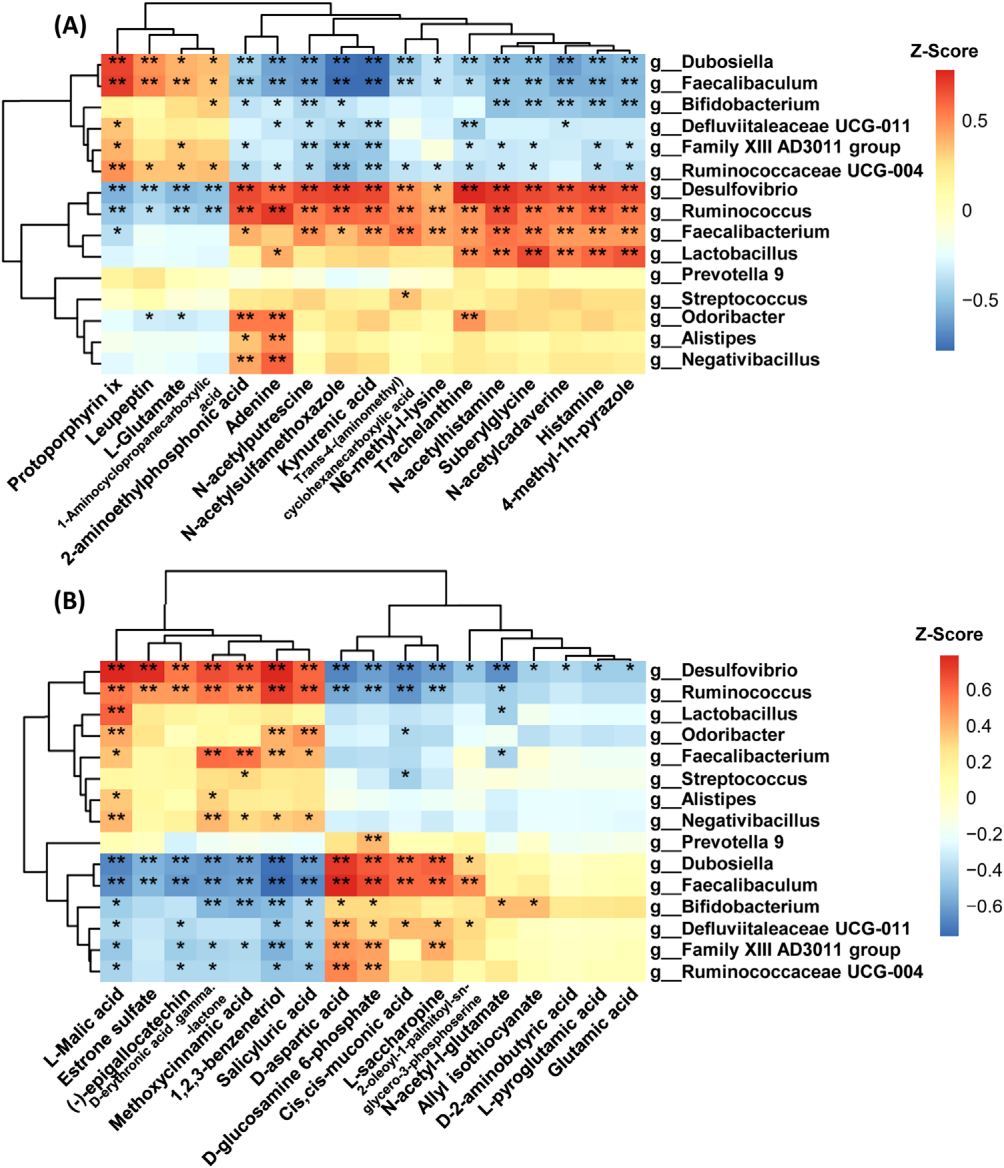
Correlation heatmap of fecal microbiota and metabolites in (A) positive and (B) negative ion mode. Con group (*n* = 9), KD group (*n* = 10), MPTP group (*n* = 9), MPTP + KD group (*n* = 10). *p < 0.05, **p < 0.01.

Furthermore, we performed UHPLC–Q‐TOF–MS/MS to measure the fecal metabolites of the four different groups. The metabolites were clearly distinguished among the four groups using discriminant analysis of partial least squares (PLS‐DA) (Figures S4A and B). The heatmap demonstrated the significantly different metabolites among four groups in positive and negative ion mode, respectively, according to the concentration profiles (Figures 5A and B and Table S1). The significant metabolic pathways affected by KD and MPTP were included in endocrine resistance, GABAergic synapse, synaptic vesicle cycle, ABC transporters, and any other metabolic pathways (Figure S4C). We also analyzed the association between gut microbiota and metabolites using the Pearson correlation analysis and demonstrated the detailed results as a heatmap (Figures 6A and B). For example, g_*Desulfovibrio* was positively correlated with histamine and salicyluric acid, whereas significantly negatively correlated N‐acetyl‐l‐glutamate, d‐aspartic acid, l‐saccharopine, and d‐glucosamine 6‐phosphate. Taken together, our results highlight KD significantly adjusted the gut microbiota and metabolites, indicating microbiota dysbiosis and related metabolites participate in the PD pathogenesis.

### 2.5 FMT from KD‐fed mice alleviated motor symptoms in MPTP‐intoxicated PD mice

**FIGURE 7:**
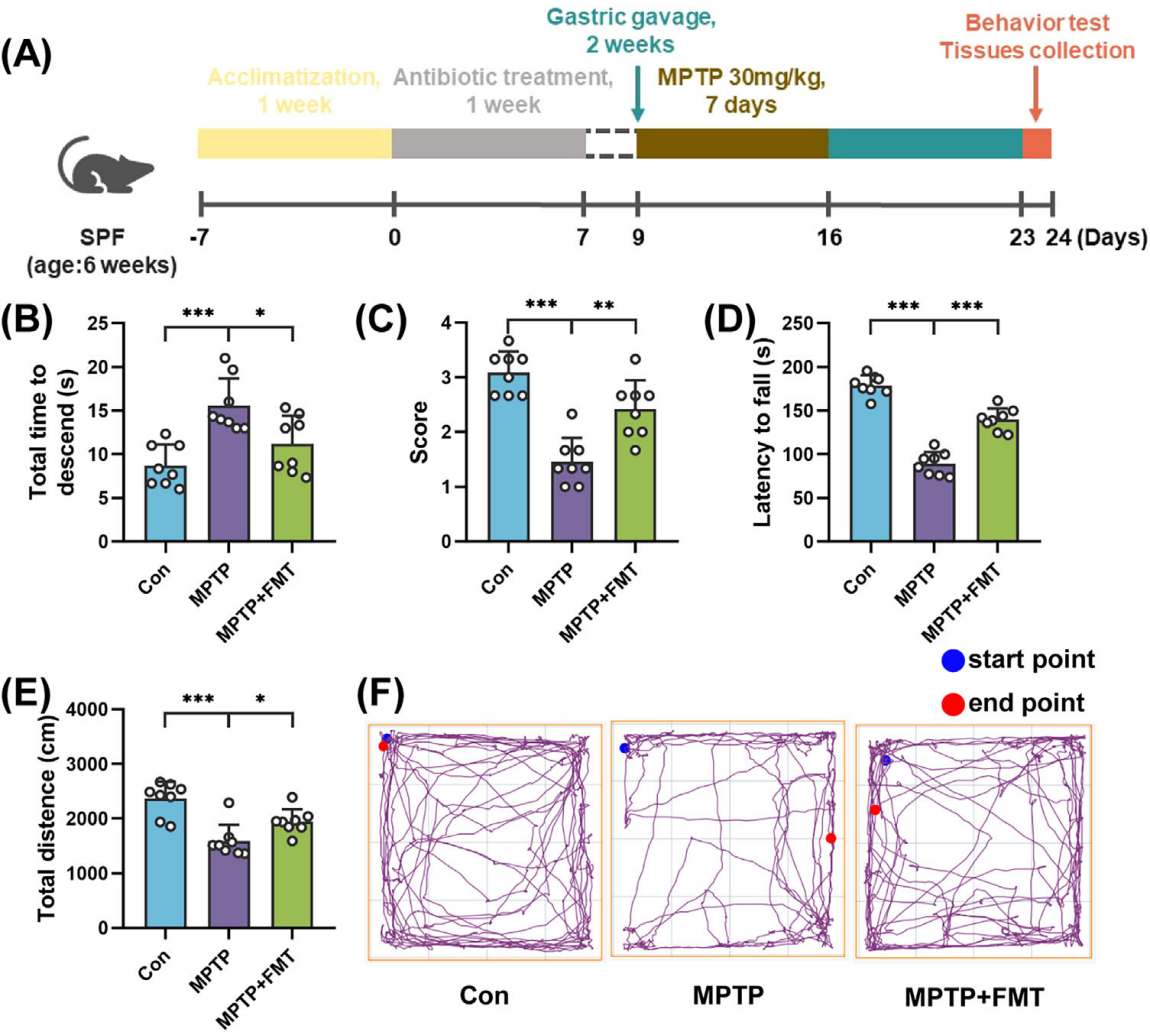
FMT from KD‐fed mice alleviated motor symptoms in MPTP‐intoxicated PD mice. (A) Schematic representation of study design. (B) Pole test, (C) traction test, (D) rotarod test, and (E, F) open field test. Data were expressed as mean ± SD, *n* = 8 mice per group, one‐way ANOVA. *p < 0.05, **p < 0.01, ***p < 0.001.

To demonstrate whether the remodeling of gut flora directly mediated the neuroprotective role of KD on MPTP‐induced PD mice, antibiotic‐pretreated PD mice received FMT from KD‐fed mice (Figure 7A). To evaluate the motor function of mice, a series of behavioral tests were conducted. Similarly, mice from the MPTP group presented behavioral deficits, including increased time to descend the pole (Figure 7B), decreased scores of traction test (Figure 7C), prolonged latency to fall from the rotarod (Figure 7D), and reduced total distance traveled (Figures 7E and F), compared with the Con group. As expected, the MPTP + FMT group significantly displayed better motor performance compared with the MPTP group, including the pole test (Figure 7B), traction test (Figure 7C), rotarod test (Figure 7D), and open field test (Figures 7E and F). Therefore, these results showed that FMT from KD‐fed mice partly improved motor dysfunction in MPTP‐induced mice.

### 2.6 FMT from KD‐fed mice restored dopaminergic neuron loss in MPTP‐intoxicated mice

**FIGURE 8:**
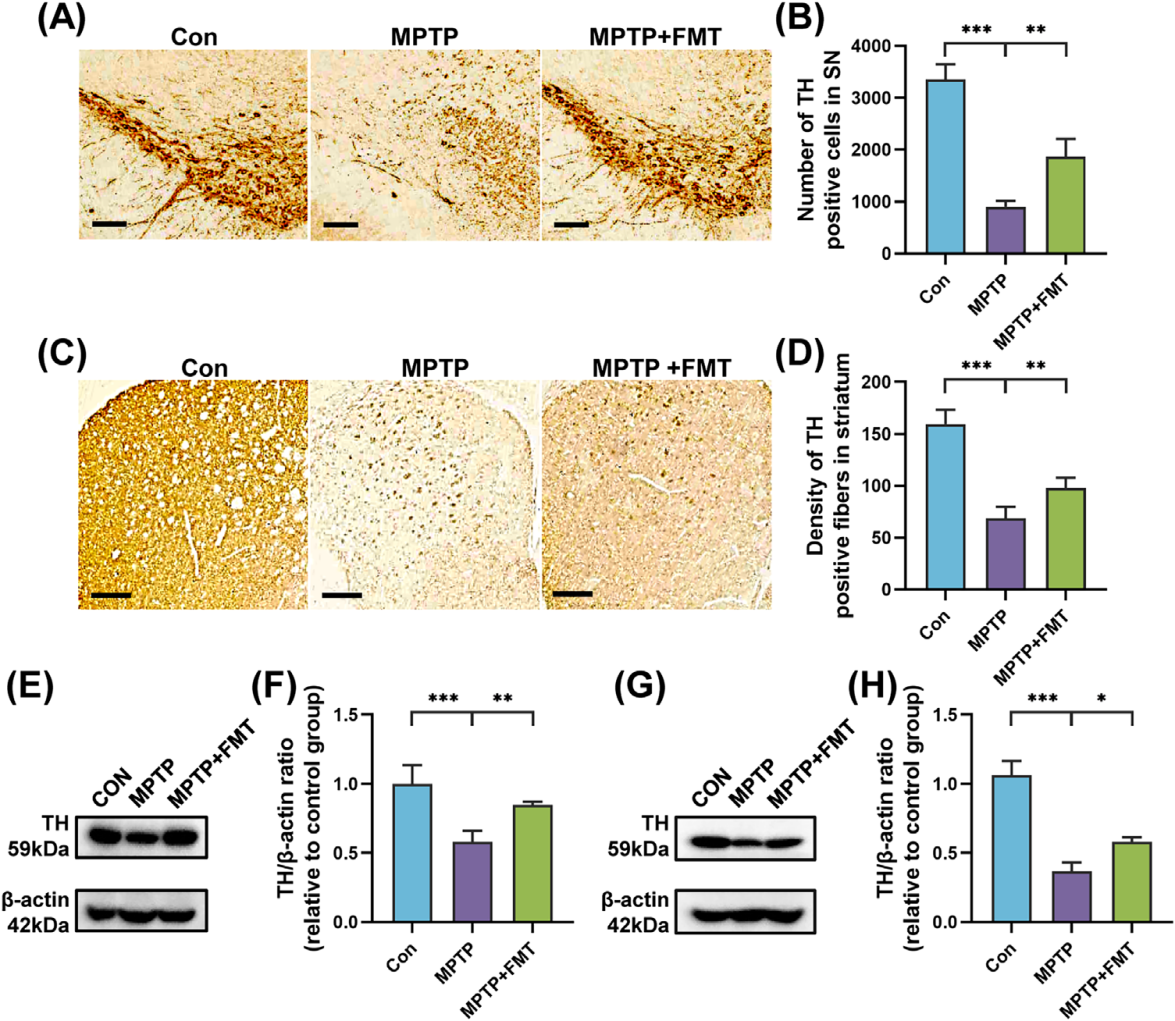
FMT from KD‐fed mice restored dopaminergic neuron loss in MPTP‐intoxicated mice. (A) Immunohistochemical staining and (B) quantitative analysis of TH in SN, scale bars = 100 μm. (C) Immunohistochemical staining and (D) quantitative analysis of TH in striatum, scale bars = 200 μm. (E) Representative western blot bands and (F) density analysis of TH in SN. (G) Representative western blot bands and (H) density analysis of TH in striatum. *p < 0.05, **p < 0.01, ***p < 0.001. Data were expressed as means ± SD, *n* = 4 mice per group, one‐way ANOVA.

Immunohistochemistry and immunoblotting for TH were performed to assess the dopaminergic neurons of each group. Compared with the Con group, TH‐positive cells in SN and TH‐positive fibers in striatum were reduced in the MPTP group (Figures 8A–D). Nevertheless, KD treatment effectively protected against the dopaminergic neuronal deficits induced by MPTP (Figures 8A–D). In addition, TH expression was both downregulated in SN and striatum of the MPTP group but remarkably up‐regulated in the MPTP + KD group (Figures 8E–H). In general, these results supported the hypothesis that gut microbiota was necessary for the protective impact of KD on MPTP‐induced PD mice.

## 3 DISCUSSION

PD is one of the most common neurodegenerative diseases and seeking a therapeutic approach to PD is urgent. In the current study, we provided evidence that 8‐week KD could attenuate motor dysfunction, dopaminergic neuron loss, and neuroinflammation in the MPTP‐induced PD male mouse model. Furthermore, KD also altered the systematic and intestinal inflammation, gut microbiota, and metabolites, suggesting that the neuroprotective effect of KD was involved in the microbiota–gut–brain axis.

In recent years, KD or exogenous KB supplements improved the motor and nonmotor symptoms in experimental and clinical research, which was consistent with our findings. For example, KD for 8 weeks was sustainable and safe and could alleviate the motor and nonmotor symptoms of PD patients in a randomized, controlled trial.^29^ Coincidentally, KD for 3 months improved the voice quality in PD patients assessed by the standard voice test.^30^ A ketone ester drink promoted endurance exercise performance in PD patients in a randomized, placebo‐controlled study.^31^ Moreover, BHB prevented dopamine uptake, dopaminergic neuron degeneration, motor function impairment, and neuroinflammation in lipopolysaccharide (LPS)‐induced PD models in vivo and in vitro. Subsequent molecular mechanism experiments demonstrated the beneficial effect was involved with G‐protein‐coupled receptor 109A modulated by the nuclear factor‐κB (NF‐κB) pathway.^28^ Exogenous BHB infusion with the pump subcutaneously modulated the complex II‐dependent mechanism, leading to enhanced ATP production, mitochondrial respiration, and motor function in MPTP‐intoxicated PD mouse model.^32^ Exogenous octanoic acid supplement, one of the fatty acids regarded as the main components of KD significantly ameliorated the depletion of dopamine and mitochondrial dysfunction in striatum of MPTP mice by regulating the mitochondrial metabolism.^33^ Interestingly, it has been shown that KD has a sex‐specific effect on the central nervous system. A recent clinical study confirmed KD improved seizure control in female patients, compared with male patients.^34^ Another study showed KD significantly improved seizure control in both male and female mice models of the epileptic Kcna1‐null mouse, while female mice fed with KD lived longer than the male counterparts.^35^ Compared with the male mice, KD promoted sociability and inhibited repetitive behavior in the epileptic female mice.^36^ The mechanisms behind this sex difference of KD need further research.

KD markedly regulated gut dysbiosis and participated in the pathogenesis of neurological diseases.^25^, ^37^ In agreement with previous studies, dysbiosis was observed in an MPTP mouse model of PD in our study and KD regime partly reversed the alteration of gut microbiota.^38^, ^39^ In the FMT experiment, we found MPTP administration generated neurotoxicity in mice pretreated with an antibiotic cocktail. However, another previous study found that a 14‐consecutive‐day antibiotic cocktail inhibited neurotoxicity in the acute MPTP mouse model of PD. Different antibiotic cocktail administration and MPTP model inductions might contribute to the conflicting results.^40^

Consistent with previous studies, we found the level of *Akkermansia* increased after the KD intervention.^26^ Compared with the healthy group, the relative abundances of *Desulfovibrio, Ruminococcus*, and *Alistipes* were increased in PD patients and models.^41^, ^42^, ^43^ KD treatment reduced the MPTP‐induced abundances of *Desulfovibrio, Ruminococcus*, and *Alistipes*, although there was no significant alteration in *Alistipes*. The *Desulfovibrio* generated LPS and hydrogen sulfide, which triggered inflammation, oxidative stress, and αSyn oligomers and aggregates.^44^ A clinical study revealed *Desulfovibrio* bacteria was positively correlated with the severity of PD based on the Hoehn‐Yahr staging progression.^45^ As a well‐known proinflammatory microbe, *Ruminococcus* could contribute to mucin degradation and promote intestinal permeability leading to aggravating inflammation.^46^, ^47^ In addition, *Ruminococcus* involved dopaminergic metabolism via suppressing TH activity.^48^ *Alistipes* was associated with inflammation, especially in colorectal cancer and depression.^49^ Though several studies reported its increased relative abundance in PD patients and related animal models, the interaction between *Alistipes* and PD needed to explore.^50^ Although *Lactobacillus* was known as one of the proverbial probiotics with an anti‐inflammatory effect, the concentration of *Lactobacillus* was elevated in PD patients.^12^, ^51^ KD treatment effectively decreased the level of *Lactobacillus* in agreement with previous studies, indicating more in‐depth studies were required to investigate the relationship among *Lactobacillus*, KD, and PD progression.^52^, ^53^ Intriguingly, we found an increase of *Citrobacter* in MPTP‐induced mice and the relative abundance of *Citrobacter* was reduced after the KD treatment. As a murine pathogen, *Citrobacter* motivated intestinal inflammation and colitis.^54^ However, few data have elaborated the effect of *Citrobacter* on PD pathogenesis. As shown in the results, *Dubosiella* was decreased in response to the MPTP‐induced toxicity, but KD significantly reversed the decrease. Notwithstanding few studies exploring the impact of *Dubosiella* on PD, previous evidence substantiated that *Dubosiella* might be a protective bacterium. *Dubosiella* appeared to play an anti‐inflammatory role due to the negative correlation with proinflammatory cytokine in the DSS‐induced mouse model of colitis.^55^ In general, our results revealed that KD treatment protected the MPTP‐induced PD mice via maintaining gut microbiota homeostasis.

Moreover, we analyzed global untargeted metabolomics of fecal samples from four groups and described a general outline of the metabolite alternation. Manifold‐significant differential fecal metabolites were identified, including inflammation and disease‐related metabolites. For example, the level of fecal histamine and N‐acetylputrescine were elevated in MPTP‐induced mice relative to the Con group and were reduced in the MPTP + KD group. Previous evidence supported that histamine level was increased in the mucosa for intestinal inflammatory disease patients.^56^ A recent clinical study revealed N‐acetylputrescine in plasma was higher in PD patients than the healthy control and was observably associated with scores of Unified Parkinson's Disease Rating Scale motor section, a test assessing PD severity.^57^ As a recognized immune response modulator, microbiota‐associated histamine induced by gut dysbiosis modulated intestinal inflammation via inflammasome and IL‐18 signaling pathways.^58^ Histamine caused the increased blood–brain barrier permeability through the endothelial H2 receptors.^59^ The histamine levels in SN and striatum were significantly elevated both in the patients with PD and in 6‐hydroxydopamine (6‐OHDA)‐induced rat model.^60^ It has been proved that histamine negatively regulates the release of dopamine in striatum. The direct interaction between dopamine receptor and histaminergic H3 receptor has been clearly observed in vivo and in vitro.^61^ Histamine facilitated microglial phagocytosis by activating H1 receptor and generated reactive oxygen species by activating H1 receptor and H4 receptor in microglial cells. Furthermore, blocking H1 receptor protected histamine‐induced dopaminergic neuron death in adult mice.^62^ The KD intervention might improve the PD pathological process by suppressing the histamine via the brain–gut axis. However, KD administration improved the low expression of d‐aspartic acid in response to MPTP toxicity. d‐Aspartic acid acted as an endogenous neurotransmitter that exerted a neuroprotective effect on neurogenesis and neuropathologies.^63^ Collectively, our data demonstrated the anti‐inflammatory effect of KD on PD mice was distinctly correlated with the alteration of metabolites.

**FIGURE 9:**
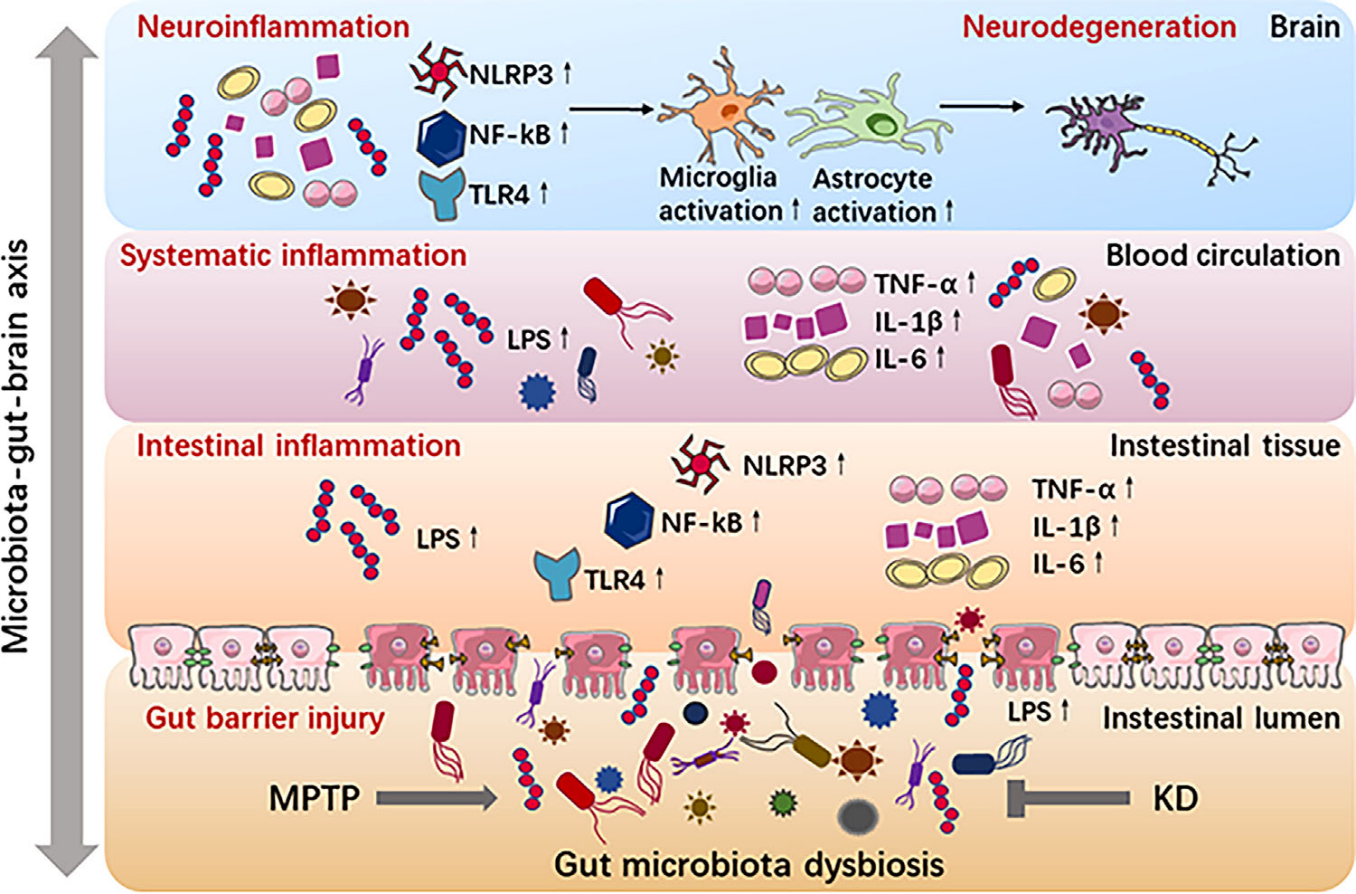
KD administration played a neuroprotective role in neurodegeneration and neuroinflammation in MPTP‐intoxicated mice via the microbiota–gut–brain axis (drawn with Science Slides and Power Point).

Our current findings revealed MPTP toxicity generated microbiota dysbiosis, intestinal inflammation and gut barrier injury. Subsequently, the increased proinflammatory cytokines and molecules leaked into the circulatory system, leading to systematic inflammation. Finally, neuroinflammation was triggered in SN and striatum, which caused the development of PD‐related pathology. Nevertheless, KD could protect MPTP‐induced PD mice by inhibiting inflammation through the diet–gut microbiota–brain axis (Figure 9). We hypothesized that the process might involve the Toll‐like receptor 4 (TLR4), NF‐κB, or NOD‐like receptor family pyrin domain containing 3 (NLRP3) inflammasome signaling pathway. However, the current study had some limitations. First, we did not investigate the potential mechanisms of the anti‐inflammatory effect of KD on gut bacteria and metabolites. A previous study has reported that gut microbiota reestablishment inhibited inflammation and decreased intestinal barrier permeability and then protected PD mice by the TLR4/NF‐κB pathway.^38^ Our other study has revealed that the BHB alleviated pyroptosis and neuroinflammation in MPTP‐induced mouse model of PD through modulating STAT3/ NLRP3 signaling pathway.^64^ It is highly relevant for ascertaining the underlying mechanisms of KD in anti‐inflammation, which deserves further research in future.^65^ Second, we only described the KD‐dominated global alteration of gut microbiota and metabolites and investigated the overall neuroprotective effect on PD pathogenesis. Thus, metagenomics or other precise detecting approaches are needed to determine one or several pivotal bacteria or metabolites that mediated the inflammation and anti‐Parkinsonian activity.^66^ Finally, our findings were primarily based on the MPTP‐induced male mouse model of PD, one of the most common PD animal models. Several generally accepted animal models of PD include neurotoxin‐induced models (MPTP, 6‐OHDA, paraquat, and rotenone) and transgenic animal models (SNCA and PINK1).^67^ Although the MPTP model can availably replicate dopaminergic neuron damage of PD, the MPTP mouse model does not generate the formation of LBs, which is the vital neuropathological feature of PD. The severity of nigrostriatal degeneration in MPTP mouse model is associated with the dose of MPTP.^68^, ^69^ Thus, the effect and safety of KD under different PD models and patients remained supported by bountiful experimental and clinical evidence in future.

In summary, these data provided crucial insights into the protective effect of KD on an MPTP‐intoxicated mouse model of PD, suggesting the process was potentially mediated by the diet–gut microbiota–brain axis. KD might be a potential strategy to prevent the onset of PD as well as slow the pathogenic processes of PD. Meanwhile, we emphasized the significance of the microbiota–gut–brain axis in the pathogenic processes of PD, implying that it could become a new therapeutic candidate for the treatment of PD.

## 4 MATERIALS AND METHODS

### 4.1 Animals, group design, and treatment

Male C57BL/6 mice aged 6 weeks (18 ± 2 g) of specific pathogen‐free (SPF) grade were provided by SPF (Beijing) Biotechnology Co., Ltd., China. Sex differences have been shown to affect the progression of PD.^70^ Due to the neuroprotective role of sex hormones (estrogen and progesterone) and sex differences in mitochondrial functionality, male mice showed increased susceptibility to the neurotoxic role of MPTP.^71^, ^72^, ^73^ Therefore, male mice were selected for this study. Mice were kept individually in ventilated cages under a standard housing condition of relative humidity and controlled temperature with a 12 h light/dark schedule and fed standard water and rodent chow food ad libitum.

There were two parts to the current study design (Figures 1A and 7A). First, animals were randomly divided into four groups (*n* = 10 mice per group): the Con group (normal control diet and 0.9% saline); the KD group (KD and 0.9% saline); the MPTP group (normal control diet and MPTP); and the MPTP + KD group (KD and MPTP). The KD contained 90.50% kcal fat and <0.50% kcal carbohydrate (TD.96355; Harlan Teklad Research Diet, Madison, USA) and the normal control diet contained 11.85% kcal fat and 65.08% kcal carbohydrate, which detailed formula of these diets were shown in Figure 1B. According to diet formula, the energy densities of the normal control diet and KD were 3.4 and 6.7 kcal/g, respectively. All mice were assigned to either the KD or the normal control diet for 8 weeks and body weights and food intakes of mice were recorded daily. From day 39 to 41, all mice received behavioral test pre‐training for 3 consecutive days. From day 42 to 48, mice were administered MPTP (30 mg/kg, intraperitoneally injection; Sigma, USA) for 7 days to develop a PD mouse model, as reported previously.^43^, ^64^ After a series of behavior tests, feces, blood samples and tissues were collected on day 56. One mouse in the MPTP group died during the MPTP administration and then the relevant data were excluded. Because of the poor quality of measurements, one sample in the Con group was excluded for fecal metabolomics.

Second, mice received antibiotic pretreatment by daily oral gavage in 200 μl for 7 consecutive days (from day 0 to 6) (Figure 7A). The antibiotics cocktail consists as follows: streptomycin 2 g/L, metronidazole 1 g/L, neomycin 1 g/L, ampicillin 1 g/L, gentamycin 0.5 g/L, and vancomycin 0.5 g/L.^74^, ^75^, ^76^ After 2 days of cessation, animals were randomly divided into three groups (*n* = 8 mice per group): the control group (0.9% saline and vehicle); the MPTP group (MPTP and vehicle); and the MPTP + FMT group (MPTP and FMT treatment). The MPTP group and MPTP + FMT group mice were treated with MPTP (from day 9 to 15). In addition, the MPTP + FMT group mice were treated with FMT using feces from the KD group mice for 2 weeks (from day 9 to 22). The Con group and MPTP group mice were treated with an equal volume of vehicle (0.9% sterile saline solution). In brief, fresh fecal pellets from the KD group mice were collected and instantly placed into 0.9% saline solution (200 mg: 2 ml) and homogenized. After the centrifugation (1000 *g*, 4°C, 5 min), the suspension was extracted and stored until administration. Each recipient mouse received the prepared suspension (200 μl) via gavage once a day for 14 consecutive days.^38^, ^76^, ^77^ Finally, the behavior tests were conducted, and tissues were collected on day 23.

### 4.2 Behavioral testing

The motor function of MPTP‐induced mice was evaluated using four behavioral tests performed on all animals, each trial being 1 h apart. We executed the rotarod test to measure motor coordination through an automatic rotarod apparatus. Animals were placed separately on the rotating rod which accelerated smoothly from 0 to 40 rpm within a 300 s period. The time mice remained on the rotating rod, namely latency to fall, was recorded. Each mouse underwent the test three times at 1 h intervals and the average latency was calculated. Other experimental protocols of the open field test, traction test and pole test have been reported previously.^64^

### 4.3 Immunohistochemistry, immunofluorescence, and image analysis

In brief, the mouse brain was embedded in paraffin and sliced into coronal sections (5 μm) of striatum and SN. Paraffin sections were deparaffinized, hydrated, antigen retrieved, incubated with H_2_O_2_ and blocked with serum.

For immunohistochemistry experiments, coronal sections were incubated with the primary antibody anti‐TH (Servicebio, GB11181) at 4°C overnight and the secondary antibody at room temperature for 2 h. Slides were visualized by diaminobenzidine staining as the chromogen and observed by a light microscopy (Nikon E100, Japan).

For immunofluorescence experiments, coronal sections were incubated with the primary antibody (anti‐IBA‐1; Servicebio, GB11105; anti‐GFAP; Servicebio, GB11096) at 4°C overnight and secondary antibody at room temperature for 1 h. Nuclei was stained by 4′,6‐diamidino‐2‐phenylindole solution. Slides were visualized and observed by a fluorescence microscope (Nikon Eclipse C1, Japan).

### 4.4 Western blot analysis

The protocols of western blot analysis have been reported previously.^64^ Briefly, quantified proteins (20 μg) were separated using 10% sodium dodecyl sulfate polyacrylamide gel electrophoresis. Separated proteins were transferred to polyvinylidene fluoride (PVDF) membranes (Millipore, USA). The PVDF membranes were incubated with the primary antibodies (anti‐TH; Abclonal; A0028 and anti‐β‐actin; Servicebio, GB15001) at 4°C overnight and HRP‐conjugated secondary antibodies (Abclonal; AS014 and AS003) at room temperature for 1 h. The blots were detected by gel image system and quantified by ImageJ software.

### 4.5 ELISA

According to the manufacturer's instructions, IL‐1β and TNF‐α in striatum, colon, and plasma were measured by ELISA kits (CSB‐E08054m and CSB‐E04741m, respectively; Cusabio, China). IL‐6 concentrations in striatum, colon and plasma were detected using an ELISA kit (H007‐1; Jiancheng Bio, Nanjing, China). Following the instructions recommended by the manufacturer, BHB concentrations in striatum and plasma were determined using the Mouse BHB ELISA kit (H169; Jiancheng Bio, China).

### 4.6 16S rDNA microbiota profiling and bioinformatics analysis

Fecal pellets defecated from each mouse were instantly collected and put into a sterile Eppendorf tube. Then the feces flash‐frozen and stored at −80°C until administration. Microbial DNA was extracted from feces by the Magnetic Soil And Stool DNA Kit (TIANGEN, China). The concentration and purity of DNA were measured by gel electrophoresis and spectrophotometer (Nanodrop 2000; Thermo Scientific). After PCR products quantification and purification and library preparation, the RNA sequencing library was detected by an Illumina MiSeq platform (Illumina, USA).^78^

Raw sequencing data were merged and filtered using FASTQ software. The operational taxonomic units (OTUs) were generated through clustering qualified reads with similarities over 97% using the UPARSE software package. Then, the representative sequences for each OTUs were annotated at taxonomic levels. Alpha diversity indices, including Chao1 and Ace, were computed for each sample using the QIIME software. Beta diversity was calculated and presented using PCoA and ANOSIM. The functional analysis of KEGG pathways was performed. Furthermore, comparisons of relative abundances of microbiota among different groups and Pearson correlation analysis were performed using R (V4.1.3).^79^, ^80^

### 4.7 Untargeted metabolomics analysis

A precooled solution (methanol, acetonitrile and water) was added to fecal samples. After the mixture, sonication, precipitation, and centrifugation, the supernatant was collected and dried in vacuum. For the UHPLC–Q‐TOF–MS/MS analysis, the dried supernatant was redissolved with 100 μl solution (acetonitrile: water). After mixture vortexing and centrifugation, the supernatant was extracted for detection using the UPLC system coupled with the mass spectrometer according to the manufacturer's instructions.^78^, ^81^ All raw data were converted into MzML format and then metabolites were identified and quantified using the XCMS program. Multivariate statistical analysis, such as PLS‐DA, and discriminant analysis of orthogonal partial least squares (OPLS‐DA), were performed by soft independent modeling of class analogy software (V14.1).^79^

### 4.8 Statistical analysis

All data were analyzed by GraphPad Prism for Windows (V 8.0 GraphPad Software Inc., La Jolla, CA, USA) and R package for Windows (V4.1.3; R Foundation for Statistical Computing, Vienna, Austria), and then the results were expressed as the mean ± standard deviation (SD). The Shapiro–Wilk test was conducted to detect data normality. One‐way or two‐way ANOVA with Turkey's or Bonferroni's post hoc test was performed for normally distributed data analysis. Kruskal–Wallis *H* test with Dunn's post‐hoc test was performed for non‐normally distributed data analysis. The *p* value < 0.05 was considered statistically significant.

## AUTHOR CONTRIBUTION

Z. J. and Z. W. initiated and conceived the project. Z. J., X. W., H. Z., J. Y., and P. Z. carried out experiments. Q. Y. performed data analysis. Z. J. and X. W. wrote the manuscript. Z. W. and Q. Y. advised the experiment and revised the article. All authors revised the manuscript and approved the final version.

## CONFLICT OF INTEREST STATEMENT

Author Jian Yin is an employee of Jinan Guo Ke Medical Technology Development Co. Ltd., and there are no potential relevant financial or nonfinancial interests to disclose. The other authors have no conflicts of interest to declare.

## ETHICS STATEMENT

All animal studies and experimental procedures were approved by the Ethics Committee of Chinese People's Liberation Army of China General Hospital (No. 2021‐X17‐80) and were performed according to the guidelines of the Institutional Animal Care and Use Committee of the hospital. All efforts were made to minimize animal suffering and to reduce the overall number of animals used.

## Supporting information

Supporting Information

Supporting Information

## Data Availability

The raw data that support the findings of this study have been deposited into the CNGB Sequence Archive (CNSA) of China National GeneBank DataBase (CNGBdb) with accession number CNP0003614 (16S rRNA Gene Sequencing) and CNP0003610 (Metabolomics). All other data needed to evaluate the conclusions in the manuscript are available from the corresponding author upon reasonable request.
