# Supplementary material for: Ketogenic diet protects MPTP‐induced mouse model of Parkinson's disease via altering gut microbiota and metabolites
Source: MedComm (2020). 2023 May 16;4(3):e268. doi: 10.1002/mco2.268 (PMC10186339; doi:10.1002/mco2.268)
Supplement: Supplementary file 1 — Supporting Information [file MCO2-4-e268-s002.doc]

Supplementary material for

**Ketogenic Diet Protects MPTP-induced Mouse Model of Parkinson's Disease via Altering Gut Microbiota and Metabolites**

Ziying Jiang^1^, Xinyu Wang^2^, Haoqiang Zhang^3^, Jian Yin^4^, Peiqing Zhao^5^, Qingqing Yin^2*^, Zhenfu Wang^1*^

**Authors’ affiliations:**

^1^Department of Geriatric Neurology, The Second Medical Center & National Clinical Research Center for Geriatric Disease, Chinese PLA General Hospital, Beijing, 100853, China.

^2^Department of Geriatric Neurology, Shandong Provincial Hospital Affiliated to Shandong First Medical University, Jinan 250021, Shandong, China.

^3^Department of Endocrinology, The First Affiliated Hospital of USTC, Division of Life Sciences and Medicine, University of Science and Technology of China, Hefei 230001, Anhui, China.

^4^CAS Key Lab of Bio-Medical Diagnostics, Suzhou Institute of Biomedical Engineering and Technology, Chinese Academy of Sciences, Suzhou 215163, Jiangsu, China; Jinan Guo Ke Medical Technology Development Co. Ltd., Jinan, Shandong, China.

^5^Center of Translational Medicine, Zibo Central Hospital Affiliated to Binzhou Medical University, Zibo 255036, Shandong, China.

***Correspondence to:**

Zhenfu Wang

Address: Department of Geriatric Neurology, The Second Medical Center & National Clinical Research Center for Geriatric Disease, Chinese PLA General Hospital, No. 28 Fuxing Road, Beijing 100853, China

Email: [zhenfuw@sina.com](mailto:zhenfuw@sina.com).

Qingqing Yin

Address: Department of Geriatric Neurology, Shandong Provincial Hospital Affiliated to Shandong First Medical University, No. 324 Jingwuweiqi Road, Jinan 250021, Shandong, China.

Email: yinqingqing@sdfmu.edu.cn.


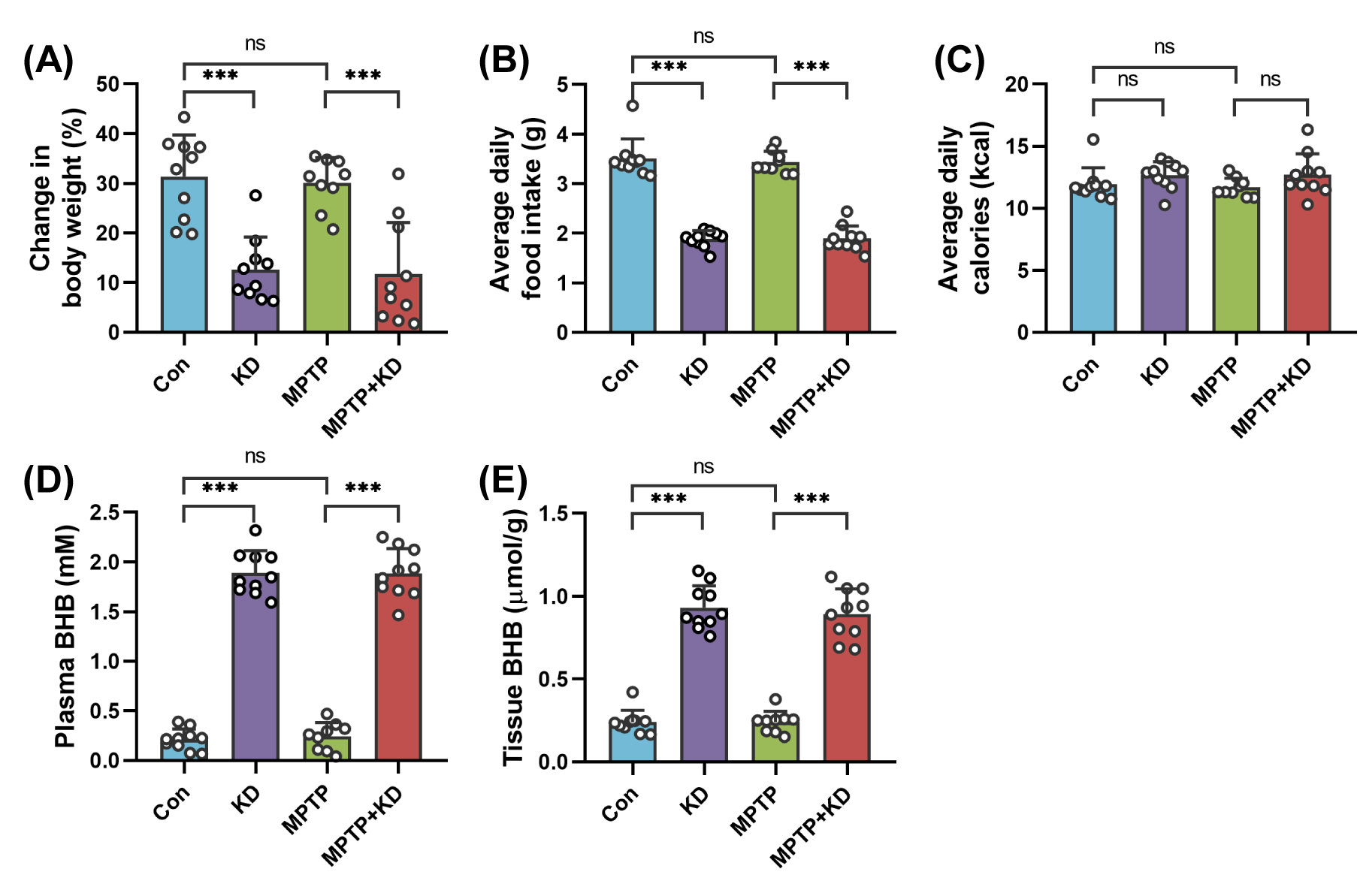


**Figure S1. Effect of KD on body weights and BHB levels in MPTP-intoxicated mice.**

(A) Body weight change compared to baseline after 8 weeks of dietary intervention (MPTP, *F*_1, 35_=0.183, *P* = 0.671; KD, *F*_1, 35_=53.466, *P* < 0.001; MPTP × KD interaction, *F*_1, 35_=0.006, *P* = 0.939). (B) Overall mean food intake (MPTP, *F*_1, 35_ = 0.162, *P* = 0.690; KD, *F*_1, 35_ = 324.017, *P* < 0.001; MPTP × KD interaction, *F*_1, 35_=0.218, *P* = 0.644) and (C) overall mean energy intake (MPTP, *F*_1, 35_=0.072, *P* = 0.790; KD, *F*_1, 35_=4.459, *P* = 0.042; MPTP × KD interaction, *F*_1, 35_=0.130, *P* = 0.720) for 8 weeks. (D) Plasma BHB levels in mice (MPTP, *F*_1, 35_=0.047, *P* = 0.830; KD, *F*_1, 35_=747.472, *P* < 0.001; MPTP × KD interaction, *F*_1, 35_=0.079, *P* = 0.780). (E) BHB concentrations in striatum of mice (MPTP, *F*_1, 35_=0.283, *P* = 0.598; KD, *F*_1, 35_=345.996, *P* < 0.001; MPTP × KD interaction, *F*_1, 35_=0.266, *P* = 0.610). Data were expressed as mean ± SD, Con group (n = 10), KD group (n = 10), MPTP group (n = 9), MPTP+KD group (n = 10), two-way ANOVA. **P* < 0.05, ***P* < 0.01, ****P* < 0.001, and ns indicates not significant.


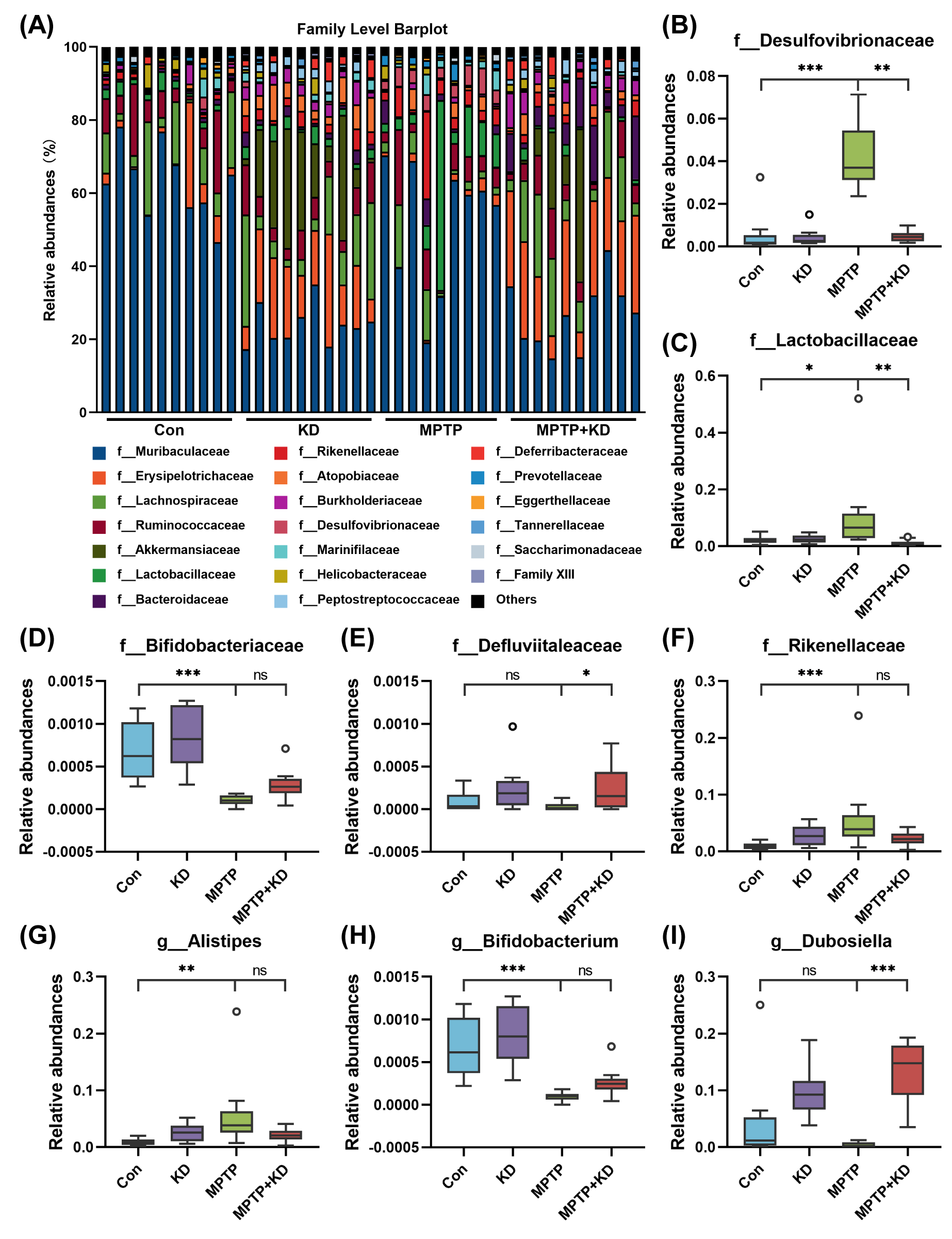


**Figure S2.** **Effect of KD on gut microbiota in MPTP-intoxicated mice.**

Barplot of relative abundance of different groups at (A) family level. Relative abundance of (B) f_*Defluviitaleaceae*, (C) f_*Lactobacillaceae*, (D) f_*Bifidobacteriaceae*, (E) f_*Defluviitaleaceae*, (F) f_*Rikenellaceae*, (G) g_*Alistipes*, (H) g_*Bifidobacterium* and (I) g_*Dubosiella* significantly changed among four different groups. Con group (n = 10), KD group (n = 10), MPTP group (n = 9), MPTP+KD group (n = 10). **P* < 0.05, ***P* < 0.01, ****P* < 0.001, and ns indicates not significant.


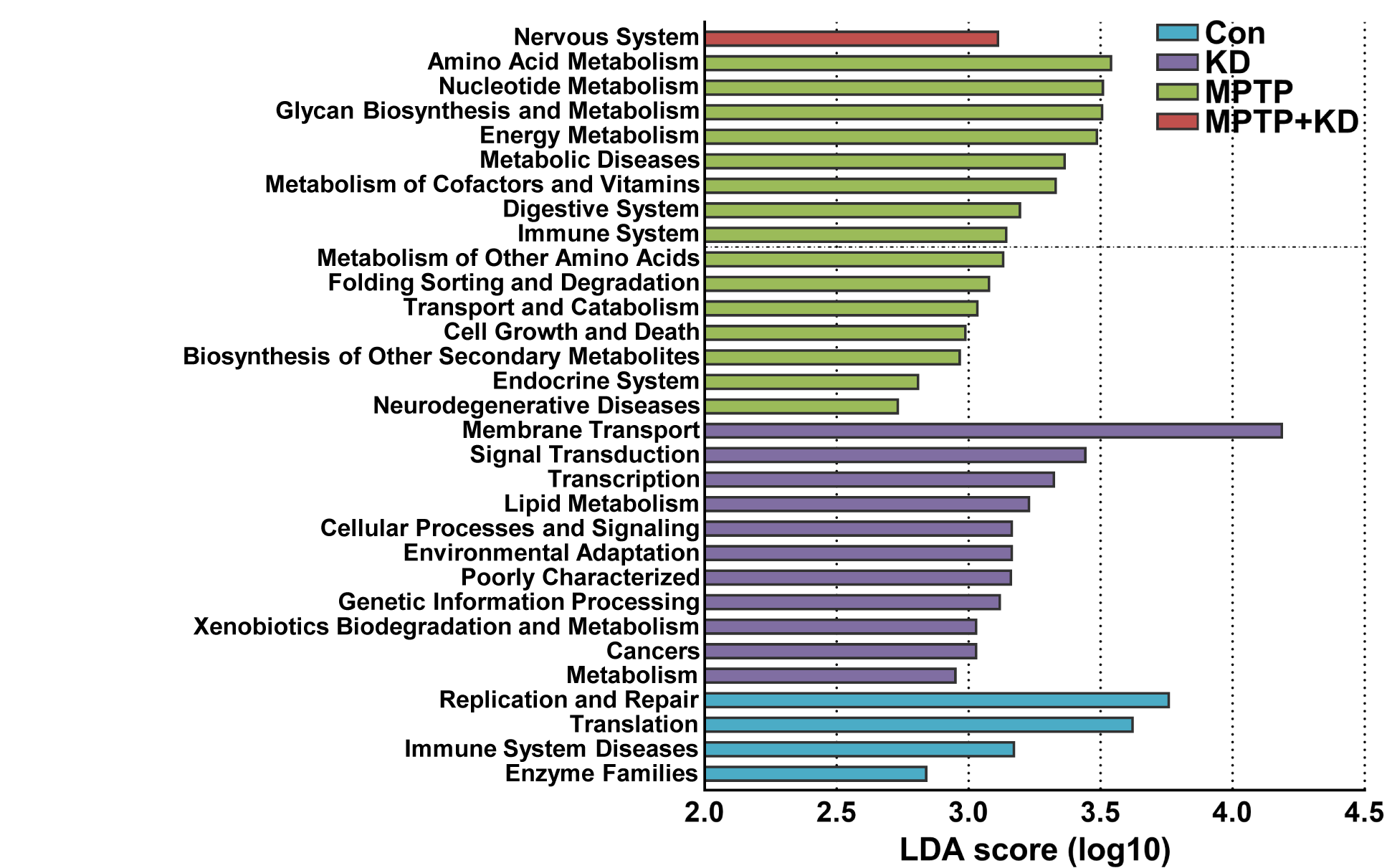


**Figure S3. Differentially enriched KEGG pathways in different groups were identified by LEfSe.**

LDA score > 2.5, *P* < 0.05. Con group (n = 10), KD group (n = 10), MPTP group (n = 9), MPTP+KD group (n = 10).


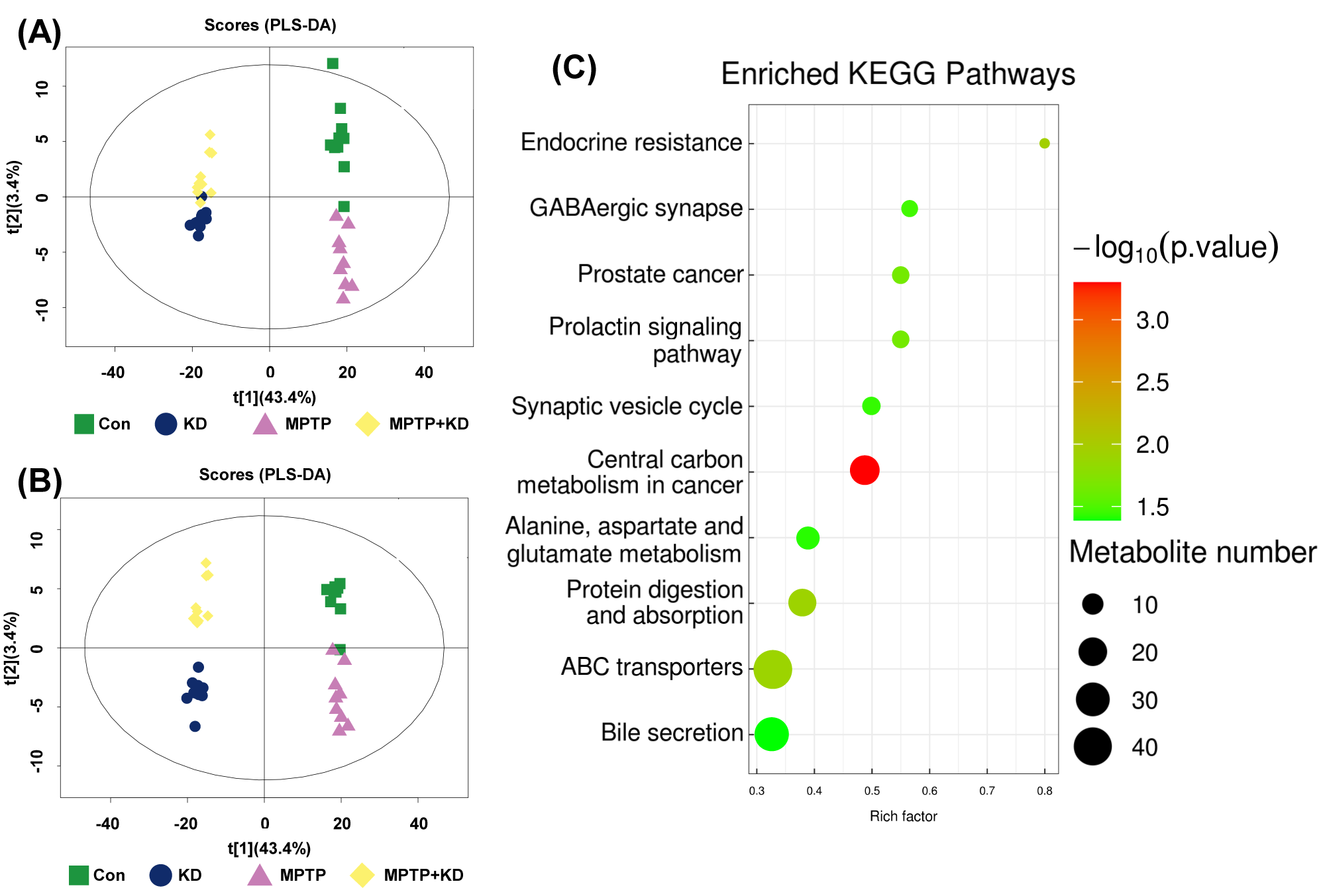
**Figure S4 Effect of KD on fecal metabolites in MPTP-intoxicated mice.**

(A, B) PLS-DA score plots derived from UHPLC-Q-TOF-MS/MS ESI (+) and ESI (−) in four groups. (C) KEGG pathway enrichment of differential metabolites among four groups. Con group (n = 9), KD group (n = 10), MPTP group (n = 9), MPTP+KD group (n = 10).
